# L-citrulline, Arginase Activity, and Nitrate As Potential Nitric Oxide-Related Markers of Subclinical Atherosclerosis in Healthy Middle-Aged Non-smokers: A Cross-Sectional Study

**DOI:** 10.7759/cureus.91720

**Published:** 2025-09-06

**Authors:** Noriyoshi Ogino, Keiki Ogino, Dai Nogimura, Kazuki Moriyasu, Masahiko Morita, Kenjiro Nagaoka

**Affiliations:** 1 Digestive Disease, University of Occupational and Environmental Health Japan, Kitakyushu, JPN; 2 Epidemiology and Public Health, Fukuoka Shin Mizumaki Hospital, Kitakyushu, JPN; 3 Nutrition, Institute of Health Sciences, Kirin Holdings Company, Fujisawa, JPN; 4 Pharmacology, College of Pharmaceutical Sciences, Matsuyama University, Matsuyama, JPN

**Keywords:** arginase, brachial-ankle pulse wave velocity, l-citrulline, nitric oxide, subclinical atherosclerosis

## Abstract

Alterations in circulating nitric oxide (NO)-related factors, which play a key role in maintaining endothelial function, are thought to precede the development of atherosclerosis. These factors are also relatively modifiable through diet and supplements. Therefore, this cross-sectional exploratory study aimed to identify blood NO-related factors associated with the risk of subclinical atherosclerosis in 124 healthy, treatment-naïve, non-smoking individuals aged 45-70 years. As a result, significant associations were observed between brachial-ankle pulse wave velocity (PWV), an indicator of arterial stiffness, and circulating levels of L-citrulline, arginase activity, and nitrate (NO_3_^-^). Specifically, circulating L-citrulline showed a negative association with PWV in non-drinkers (p = 0.007, β = -0.222). Arginase activity was positively associated with PWV among participants with a body mass index (BMI) ≤ 23 (p = 0.011, β = 0.196). Furthermore, plasma NO_3_^-^ levels exhibited a negative association with PWV in drinkers (p = 0.001, β = -0.274) and a positive association in non-drinkers (p = 0.005, β = 0.241). In conclusion, L-citrulline, arginase activity, and plasma NO_3_^-^ are potential markers of early vascular changes within subgroups of healthy middle-aged adults and may serve as targets for preventive medical approaches using diet or supplementation.

## Introduction

Atherosclerosis is a well-established precursor in most vascular diseases and accounts for 37% of deaths from noncommunicable diseases among individuals aged <70 years [1]. Effective atherosclerosis prevention requires proactive management of three major risk factors - diabetes, dyslipidemia, and hypertension [2]. However, interventions need to be initiated before the onset of these conditions, considering the sharp increase in lifestyle-related diseases. Educating people about lifestyle changes (such as reducing calorie intake, quitting smoking, limiting alcohol consumption, and promoting physical activity) is important for the prevention and management of atherosclerosis, but it is often difficult for the target population to put these changes into practice [3]. Hence, identifying objective indicators that justify early intervention is crucial, particularly in relatively healthy middle-aged populations demonstrating normal glucose tolerance, lipid profiles, and blood pressure but experiencing the early stages of atherosclerosis and vascular dysfunction.

A potential indicator involves factors influencing the bioavailability of nitric oxide (NO), given that endothelial dysfunction induced by reduced NO precedes atherosclerosis [4]. Nitric oxide synthase (NOS) from L-arginine typically produces NO, with L-citrulline as a byproduct [5]. Asymmetric dimethylarginine (ADMA), structurally similar to L-arginine, competes with NOS and inhibits NO production [6]. Arginase, another enzyme that utilizes L-arginine, produces L-ornithine and hinders NO production by competing with NOS [7]. Thus, amino acids play a role associated with NO production [8]. Factors associated with NO bioavailability may act as useful intervention indicators, considering that amino acids can be supplemented. Additionally, nitrate (NO_3_^−^) dietary intake is converted to NO via nitrite (NO_2_^−^) through enzymatic activity, stomach pH, and bacterial flora in the mouth and intestines, emphasizing the significance of NO_3_^−^ in NO production [8]. Imbalances in these factors have been observed in patients with atherosclerotic disease, but their relevance as NO bioavailability indicators in relatively healthy middle-aged individuals remains unclear. Therefore, an exploratory cross-sectional study was conducted based on the hypothesis that factors related to the bioavailability of NO reflect the early stages of atherosclerotic changes in middle-aged healthy individuals. We aimed to evaluate whether circulating NO-related factors - L-citrulline, arginase activity, and nitrate - are associated with vascular function (flow-mediated dilation (FMD) and brachial-ankle pulse wave velocity (PWV)) in healthy, treatment-naïve, non-smoking adults aged 45-70 years, and to explore subgroup-specific associations by BMI, alcohol use, and systolic blood pressure (SBP).

## Materials and methods

Subjects

In accordance with the ethical principles of the Declaration of Helsinki and the ethical guidelines for medical research involving human participants, the Research Ethical Review Committee of Kirin Holdings Company, Limited and Hikobae Medical Corporation and Tsukiji Futaba Clinic Ethics Review Committee approved this study (ethics approval number: 2021-007-3). Recruitment was publicly announced to healthy volunteers, aged 45-70 years old, who were registered in the participant pool held by the study’s contract research organization, Synapse Planning Inc. Screening tests were conducted on consecutively enrolled potential participants who provided consent by the deadline. Specifically, a lifestyle questionnaire, medical interview, and physical examination, blood biochemical testing, and pulse rate, height, weight, and blood pressure measurements were performed.

Based on these test results, the final participants were selected according to the following exclusion criteria: women who had not yet reached menopause or who had reached menopause but had menstruated within the past year; those with measured blood pressure exceeding 139 mmHg SBP or 89 mmHg diastolic blood pressure (DBP); those taking supplements or health food (including food for specified health uses and food with functional claims) or participating in such studies; those whose average daily alcohol intake exceeded 20 g; those who have a history of smoking (daily or occasional cigarette smoking in the past three months); those who received hormone replacement therapy within three months prior to study entry; diagnosed with asthma; those who exercise (more than 120 min per week); those with triglycerides above 350 mg/dL, low-density lipoprotein (LDL) cholesterol above 190 mg/dL, and HbA1c above 6.5 on the blood test for this screening; those with a cardiovascular, liver, renal, respiratory, endocrine or metabolic disease, or a history of these diseases; and those receiving medication from a physician. If the screening results lead to a diagnosis of the above diseases by the physician, the therapeutic intervention or therapeutic observation necessary is determined by the physician. Participants selected for the screening test visited the Tokyo Seaside Clinic within two weeks for a second examination. Specifically, after a medical examination to assess health status, FMD was performed to assess vascular endothelial function, and ankle-brachial index (ABI) and brachial-ankle PWV tests were performed to estimate arterial stiffness [9]. The evaluator for this measurement is blinded because they are a contracted vendor. Blood samples were then taken through peripheral venipuncture to determine ADMA, high-sensitivity C-reactive protein (hs-CRP), blood amino acids (L-arginine, L-citrulline, and L-ornithine), arginase, arginase activity, NO_2_^−^, and NO_3_^−^. All participants arrived after an overnight fast (water permitted) and refrained from alcohol, caffeine, smoking, vigorous exercise, and antibacterial mouthwash for 24 hours prior to any testing. And they were instructed to avoid eating a meal containing more than 2000 µg/g of NO_2_^−^ for dinner a day prior to the visit. These foods include pak choi, tsuma greens, ha sui imo (ryukyu), komatsuna, bok choy, garland chrysanthemum, mitsuba, celery, parsley, spinach, kohlrabi, and tatsoi.

Blood sample preparation

After blood withdrawal, the red blood cell component was completely separated from the serum using a blood collection tube with a separation gel and coagulation accelerator in a few minutes by centrifugation at 1740 ×g to prevent rapid oxidation of NO_2_^−^ to NO_3_^−^ by red blood cells. Subsequently, the serum was frozen at -80℃.

FMD, PWV, and ABI

A validated oscillometric instrument, HEM-705IT (Omron Healthcare Inc., Kyoto, Japan), was used to measure blood pressure. Longitudinal echo scans of the brachial artery were performed before and after reactive hypertension using high-resolution ultrasonography (UNEXEF-38G; UNEX, Nagoya, Japan). FMD was assessed in a temperature-controlled room (22-24°C) in the morning after an overnight fast. Participants abstained from alcohol, caffeine, smoking, vigorous exercise, and antibacterial mouthwash for 24 hours prior to testing. After 10-15 minutes of supine rest, a cuff was placed distal to the antecubital fossa and inflated to 50 mmHg above systolic pressure for five minutes. Diameter was tracked continuously, and peak dilation within 60-120 seconds post-deflation was used for FMD (%). Brachial-ankle PWV was measured (Vascular Profiler BP-203RPETM system, Omron, Japan) after ≥10 minutes of supine rest in a quiet, temperature-controlled room. Cuffs were applied to both upper arms and ankles per manufacturer protocol; participants arrived in the morning after an overnight fast. Ankle and arm blood pressure measurements were combined to produce the ABI. All physical measurements were conducted by trained medical staff.

Biochemical analyses

A latex agglutination method (JCA-BM9130, JEOL, Tokyo, Japan) was used to measure HbA1c. Recommended methods of the Japanese Society of Clinical Chemistry were used to test LDL cholesterol and triglyceride levels. The Hitachi automated analyzer Labospect LST008α (Hitachi Co., Tokyo, Japan) was integrated with the JCA-BM8000 automatic analyzer (JEOL Co., Inc., Tokyo, Japan). Standard laboratory procedures were used to test serum hs-CRP levels (JCA-BM9130, BioMajestyTM, JEOL, Tokyo, Japan). Tandem mass spectrometry analysis combined with liquid chromatography was used to determine the amino acids. ADMA was measured using the ADMA ELISA kit (Immundiagnostik AG, Bensheim, Germany) with a Versamax microplate reader (Molecular Devices, California, USA).

Arginase 1 activity

Following the manufacturer’s instructions, an ELISA kit (BioVendor Laboratory Medicine, Karasek, Czech Republic) was used to measure the serum concentration of arginase I protein. The serum arginase activity was measured in reference to a previous paper [10]. In summary, an Amicon Ultra centrifugal filter (10,000 molecular weight cut-off; Merck Millipore, Burlington, MA) was used to remove endogenous urea from serum samples. The samples were incubated with substrate buffer containing 100 mM arginine and 2 mM magnesium chloride at 37°C for 120 min. A urea assay kit (BioAssay Systems, Hayward, CA) was used to measure the amount of urea produced in accordance with the manufacturer’s instructions. Briefly, arginase was activated for 10 minutes at 55°C in the presence of manganese chloride (MnCl_2_), followed by the addition of arginine and incubation for 60 minutes at 37°C. The hydrolysis of arginine was stopped with acid, and the reaction product, urea, was measured by a colorimetric assay at 560 nm after incubation with α-isonitrosopropiophenone for 45 minutes at 100°C.

Analysis of NO_2_^−^ and NO_3_^−^

The amount of NO_2_^−^ and NO_3_^−^ in serum was measured using several modifications of a previous method [11]. Serum was deprotenized using Amincon ultrafiltration membrane (10 kDa; Merck KGaA, Darmstadt, Germany) and then analyzed for NO_2_^−^ levels with 2,3-diaminonaphthalene (DAN; Dojindo, Kumamoto, Japan). NO_3_^−^ is the value obtained by subtracting NO_2_^−^ from the value measured as NOx (NO_2_^−^ + NO_3_^−^) by NO_3_^−^ reduction to NO_2_^−^ with nitrate reductase (Sigma Aldrich, St. Louis, Missouri, USA) in the presence of nicotinamide adenine dinucleotide phosphate. The sample was mixed for five minutes in the dark, and the reaction was then discontinued by adding sodium hydroxide. A fluorescence microplate reader (Molecular Devices, Sunnyvale, CA, USA) was used with excitation at 365 nm and emission at 430 nm to detect fluorescence intensity. Additionally, NO_2_^−^ was measured by adding DAN without reduction, and nitrate was calculated by subtracting the values of NOx from NO_2_^−^.

Statistical analyses and justification of the sample size

The means ± standard deviations (95% confidence intervals, if applicable) are provided for all data. Differences in the mean values of several clinical parameters were analyzed according to age, sex, exercise habits (divided into two groups: those who did not exercise at all and those who exercised for less than 120 minutes per week), alcohol consumption (classified as drinkers ≥1 episode/month or non-drinkers <1/month), and pollen allergy using the chi-square test, unpaired t test, or Mann-Whitney U test. We investigated the relationship between FMD and PWV and NO-related measures, clinical parameters, and lifestyles using Spearman’s correlation and multiple regression analyses. To investigate significant associations between the dependent variables, FMD or PWV, and the different covariates, multiple regression analysis was performed on the entire study population using the stepwise method, followed by a stratified analysis of the significant variables obtained, stratified by their mean values. Covariates for adjustment were age, sex, BMI, FMD, PWV, ABI, SBP, hs-CRP, L-ornithine, L-arginine, arginase activity, L-citrulline, ADMA, NO_2_^−^, NO_3_^−^, alcohol consumption, and exercise. Because SBP and DBP, as well as arginase 1 and arginase activity, were strongly correlated, it was necessary to include only one variable from each pair as an explanatory variable to avoid multicollinearity. Therefore, SBP and arginase activity were selected as the explanatory variables. We assessed multicollinearity using the variance inflation factor. For multiple regression analysis, FMD, PWV, ABI, hs-CRP, L-ornithine, L-arginine, L-citrulline, arginase activity, NO_2_^−^, and NO_3_^−^ were log-transformed, and ADMA was converted to a power of 1.7, which is close to a normal distribution. Normality was confirmed using the Shapiro-Wilk test. For p < 0.05, all probability values were considered statistically significant. GraphPad Prism 5.0 for Mac (GraphPad Software, Inc., San Diego, CA, USA) and PASW Statistics 18 for Mac (SPSS Inc., Chicago, IL, USA) were used for all statistical analyses. The sample size computation was predicated on previous investigations [11] on changes in blood arginase concentration and L-arginine, which are important for NO bioavailability, in which the effect size was calculated as effect size = 0.275. For a power of β = 0.8 and a significance level of α = 0.05, the total sample size was 98. Considering a 24 (20%) dropout rate, the target number of participants after screening was set at 120. The target number of final participants was set at 120, based on the assumption that some individuals would be excluded during screening and others would drop out before completing the second visit. To account for both exclusions based on predefined criteria and participant attrition between screening and follow-up testing, we initially planned to recruit approximately 200 participants.

## Results

Study population

**Figure 1:**
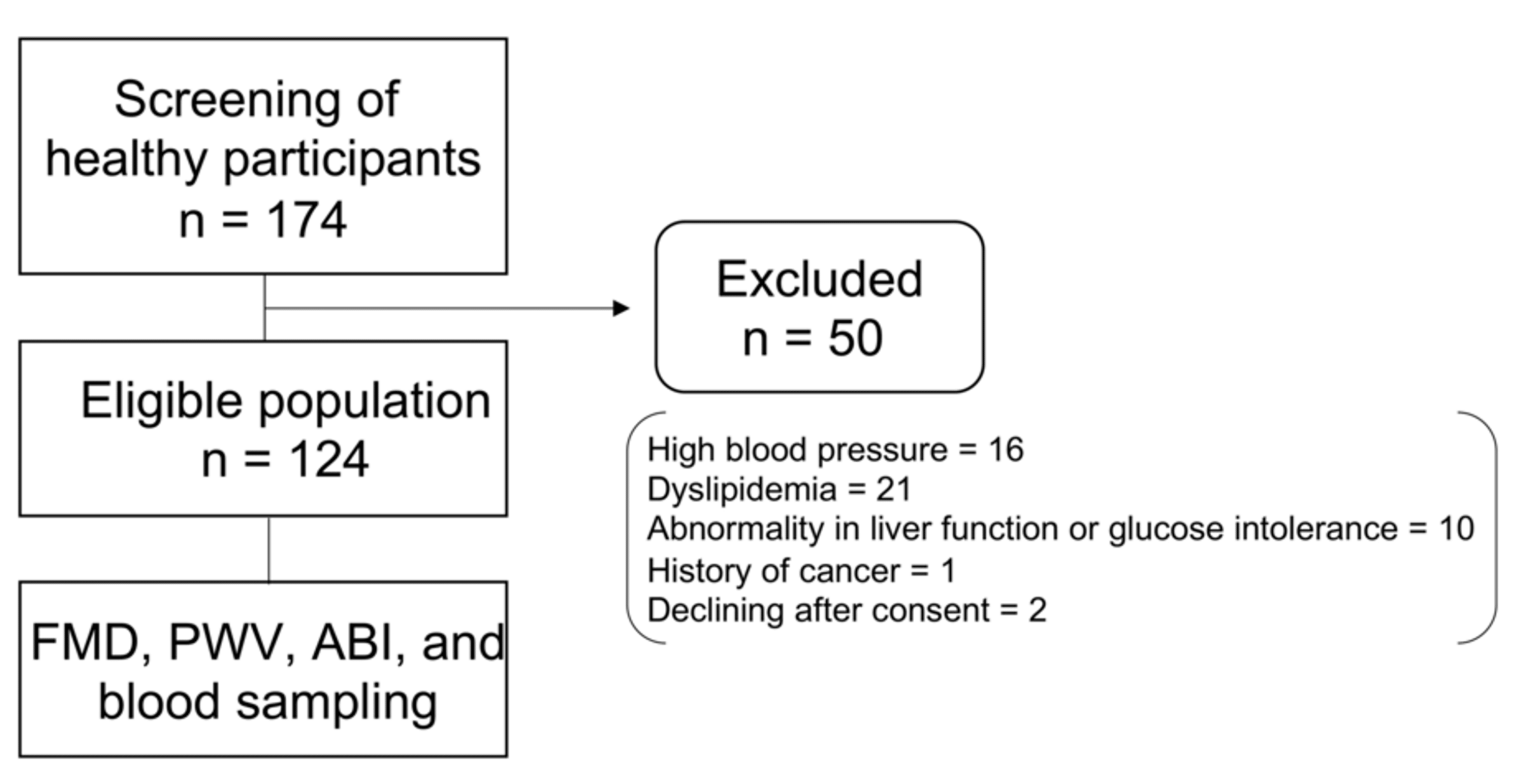
Flow chart for this cross-sectional study Of the 174 healthy participants who underwent screening tests, 50 were excluded, including 16 for high blood pressure, 21 for dyslipidemia, 10 for liver function abnormalities or glucose intolerance requiring medical intervention, one for breast cancer, and two for declining after consent. Finally, 124 individuals who met the criteria underwent flow-dependent vasodilation (FMD), brachial-ankle pulse wave velocity (PWV), ankle-branchial index (ABI), and a second blood test.

A flowchart of the selection process for the study participants is shown in Figure 1.

Initially, we planned to accept 200 entries, but the 174 apparently healthy Japanese men and women who participated in the study underwent screening tests. Then, 124 individuals who satisfied the criteria underwent FMD, PWV, ABI, and a second blood test within two weeks. A total of 50 patients were excluded: 16 for high blood pressure, 21 for dyslipidemia, 10 for abnormalities in liver function or glucose intolerance requiring medical intervention, one for breast cancer, and two for declining after consent.

Characteristics of the participants

The characteristics of the 124 individuals are shown in Table 1.

**Table 1:** Basic statistics of the 124 participants BMI: body mass index; FMD: flow-mediated dilation; PWV: pulse wave velocity; ABI: ankle-branchial index; hs-CRP: high sensitivity C-reactive protein; ADMA: asymmetric dimethylarginine; NO_2_^-^: nitrite; NO_3_^-^: nitrate; p: p-value

Variables	Total	Male	Female	p
	n = 124	n = 61	n = 63	
Age	57.72 ± 6.85	57.44 ± 7.02	57.98 ± 6.73	0.66
BMI	23.37 ± 2.79	24.13 ± 2.52	22.64 ± 2.86	0.003
FMD (%)	4.43 ± 2.89	4.39 ± 2.6	4.47 ± 3.17	0.87
PWV (m/s)	1.36 ± 0.23	1.37 ± 0.23	1.35 ± 0.22	0.80
ABI	1.15 ± 0.06	1.16 ± 0.06	1.14 ± 0.06	<0.001
Systolic blood pressure (mmHg)	120.8 ± 15.5	122 ± 13.74	119.6 ± 17.07	0.31
Diastolic blood pressure (mmHg)	75.92 ± 10.87	79.8 ± 9.8	72.16 ± 10.61	<0.001
hs-CRP (mg/dL)	0.05 ± 0.07	0.05 ± 0.06	0.05 ± 0.08	0.37
Arginase-1 (ng/mL)	6.54 ± 5.47	6.22 ± 5.34	6.84 ± 5.61	0.60
Arginase activity (mM/min)	1.01 ± 0.66	0.98 ± 0.68	1.03 ± 0.63	0.51
L-arginine (nmol/mL)	96.4 ± 17.95	99.08 ± 18.03	93.8 ± 17.62	0.09
L-citrulline (nmol/mL)	33.2 ± 5.89	33.2 ± 6.01	33.19 ± 5.83	0.75
L-ornithine (nmol/mL)	65.83 ± 13.67	68.41 ± 14.69	63.33 ± 12.21	0.06
L-arginine/ADMA ratio	204.65 ± 84.94	199.43 ± 42.06	209.7 ± 112.02	0.96
ADMA (mmol/L)	0.49 ± 0.08	0.5 ± 0.07	0.48 ± 0.08	0.06
NO_2_^-^ (mM)	0.35 ± 0.19	0.36 ± 0.17	0.33 ± 0.2	0.22
NO_3_^-^ (mM)	17.7 ± 14.59	16.44 ± 9.72	18.92 ± 18.05	0.84
Drinker	62	37	25	0.02
Exercise habit	42	23	19	0.38
Pollen allergy	20	6	14	0.16
Statistical analysis between two groups of continuous or ordinal scale variables was conducted by chi-squared test or Student's t test, or Mann-Whitney U test. Data are presented as mean ± standard deviation or number of subjects.				

Males had significantly higher BMI, ABI, and DBP values and were more likely to have a drinking habit than females. Next, Table 2 shows the results when divided by the means of age (58 years) and the average BMI (23).

**Table 2:** Basic statistics of the 124 participants in the analysis stratified by age and BMI BMI: body mass index; FMD: flow-mediated dilation; PWV: pulse wave velocity; ABI: ankle-branchial index; hs-CRP: high sensitivity C-reactive protein; ADMA: asymmetric dimethylarginine; M/F: male/female; NO_2_^-^: nitrite; NO_3_^-^: nitrate; p: p-value

Variables	Age < 58	Age ≥ 58		BMI < 23	BMI ≥ 23	
	n = 64	n = 60	p	n = 57	n = 67	p
Age	52.03 ± 3.51	63.78 ± 3.5	<0.001	57.37 ± 6.83	58.01 ± 6.92	0.60
Sex (M/F)	33	28	0.59	20	41	0.004
BMI	22.88 ± 2.64	23.91 ± 2.86	0.04	20.97 ± 1.33	25.42 ± 1.94	<0.001
FMD (%)	5.11 ± 2.85	3.7 ± 2.79	0.003	5.01 ± 3.09	3.94 ± 2.65	0.04
PWV (m/s)	1.24 ± 0.17	1.48 ± 0.22	<0.001	1.35 ± 0.25	1.36 ± 0.21	0.46
ABI	1.15 ± 0.06	1.15 ± 0.06	0.68	1.14 ± 0.06	1.16 ± 0.06	0.17
Systolic blood pressure (mmHg)	115.8 ± 14.28	126.1 ± 15.1	<0.001	116.2 ± 15.37	124.7 ± 14.64	0.001
Diastolic blood pressure (mmHg)	73.81 ± 11.98	78.17 ± 9.13	0.03	72.09 ± 10.08	79.18 ± 10.36	<0.001
hs-CRP (mg/dL)	0.05 ± 0.06	0.06 ± 0.07	0.12	0.027 ± 0.034	0.073 ± 0.083	<0.001
Arginase-1 (ng/mL)	5.99 ± 5.26	7.12 ± 5.66	0.19	5.37 ± 4.37	7.54 ± 6.11	0.003
Arginase activity (mM/min)	1.01 ± 0.66	1 ± 0.66	0.71	0.86 ± 0.55	1.13 ± 0.71	<0.001
L-arginine (nmol/mL)	95.05 ± 18.61	97.83 ± 17.26	0.39	96.68 ± 18.22	96.16 ± 17.85	0.67
L-citrulline (nmol/mL)	32.27 ± 5.49	34.19 ± 6.18	0.14	33.84 ± 6.49	32.66 ± 5.32	0.27
L-ornithine (nmol/mL)	65.74 ± 13.21	65.93 ± 14.26	0.73	63.61 ± 12.51	67.72 ± 14.41	0.08
L-arginine/ADMA ratio	211.01 ± 112.72	197.87 ± 37.2	0.92	216.92 ± 117.54	194.21 ± 38.51	0.08
ADMA (mmol/L)	0.48 ± 0.08	0.5 ± 0.08	0.13	0.48 ± 0.08	0.5 ± 0.07	0.11
NO_2_^-^ (mM)	0.33 ± 0.15	0.36 ± 0.22	0.98	0.36 ± 0.2	0.34 ± 0.17	0.68
NO_3_^-^ (mM)	16.48 ± 11.41	19.01 ± 17.3	0.66	18.96 ± 18.1	16.63 ± 10.7	0.85
Drinker	29	33	0.28	28	34	0.71
Exercise	22	22	0.70	21	21	0.52
Pollen allergy	13	7	0.19	5	15	0.05
Statistical analysis between two groups of continuous or ordinal scale variables was conducted by chi-squared test or Student's t test, or Mann-Whitney U test. Data are presented as mean ± standard deviation or number of subjects.						

The group over 58 had a significantly higher BMI, blood pressure, and PWV, and significantly lower FMD than the group under 58. The group over 23 had significantly more males; significantly higher blood pressure, hs-CRP, arginase, and arginase activity; and significantly lower FMD than the group with a BMI of <23. In this study, the participant characteristics such as male sex, older age groups, and those with high BMI are consistent with those reported for larger demographics and represent well the characteristics of middle-aged populations [12]. The correlation of BMI with arginase and hs-CRP level is also consistent with previous reports [13].

Spearman’s correlation of each variable

Tables 3-4 show the Spearman’s correlation for each variable.

**Table 3:** Spearman's correlation for each variable of the 124 participants BMI: body mass index; FMD: flow-mediated dilation; PWV: pulse wave velocity; ABI: ankle-branchial index; hs-CRP: high sensitivity C-reactive protein; ADMA: asymmetric dimethylarginine; NO_2_^-^: nitrite; NO_3_^-^: nitrate; r: the correlation coefficient; p: p-value

Variables	FMD		PWV		Arginase1		Arginase activity		ADMA		NO_2_^-^		NO_3_^-^	
	r	p	r	p	r	p	r	p	r	p	r	p	r	p
Age	-0.285	0.001	0.555	<0.001	0.114	0.21	-0.061	0.50	0.161	0.07	-0.061	0.39	0.11	0.23
Sex	-0.015	0.87	-0.024	0.79	0.048	0.60	0.060	0.51	-0.173	0.06	-0.11	0.22	-0.018	0.84
BMI	-0.14	0.12	0.181	0.04	0.261	0.003	0.258	0.004	0.208	0.02	-0.059	0.52	-0.002	0.99
FMD	-	-	-0.311	<0.001	0.027	0.77	0.015	0.87	0.065	0.47	-0.009	0.92	-0.183	0.04
PWV	-0.311	<0.001	-	-	0.065	0.48	0.043	0.63	0.14	0.12	-0.072	0.42	<0.001	1.00
ABI	0.003	0.98	-0.021	0.81	-0.047	0.61	-0.033	0.72	0.111	0.22	0.023	0.80	-0.045	0.62
Systolic blood pressure	-0.124	0.17	0.685	<0.001	0.03	0.74	-0.014	0.88	0.218	0.02	0.004	0.97	-0.028	0.76
Diastolic blood pressure	-0.092	0.31	0.587	<0.001	0.002	0.99	-0.012	0.89	0.195	0.03	0.078	0.39	-0.029	0.75
hs-CRP	-0.042	0.65	0.179	0.047	0.123	0.18	0.121	0.18	0.143	0.11	-0.194	0.03	-0.016	0.86
Arginase 1	0.027	0.77	0.065	0.48	-	-	0.722	<0.001	0.045	0.62	-0.142	0.12	-0.039	0.67
Arginase activity	0.015	0.87	0.043	0.63	0.722	<0.001	-	-	-0.016	0.86	-0.251	0.005	-0.026	0.77
L-arginine	0.070	0.44	0.013	0.88	-0.02	0.82	-0.109	0.23	0.253	0.005	0.177	0.05	-0.063	0.49
L-ornithine	0.059	0.52	0.078	0.39	0.297	<0.001	0.211	0.02	0.250	0.005	0.114	0.21	-0.168	0.06
L-citrulline	0.131	0.15	-0.039	0.67	0.067	0.46	-0.026	0.77	-0.148	0.11	0.164	0.07	-0.025	0.78
ADMA	0.065	0.47	0.140	0.12	0.045	0.62	-0.016	0.86	-	-	0.151	0.09	-0.034	0.71
L-arginine/ADMA	0.043	0.64	-0.105	0.25	-0.063	0.49	-0.14	0.12	-	-	0.075	0.41	-0.075	0.41
NO_2_^−^	-0.009	0.92	-0.072	0.42	-0.142	0.12	-0.251	0.005	0.151	0.09	-	-	-0.1	0.27
NO_3_^−^	-0.183	0.04	0.001	1.00	-0.04	0.67	-0.026	0.77	-0.034	0.71	-0.1	0.27	-	-
Alcohol consumption	-0.22	0.02	0.275	0.002	-0.066	0.47	-0.084	0.36	0.085	0.35	0.001	0.11	0.145	0.11
Exercise	0.021	0.82	0.047	0.60	-0.05	0.58	0.015	0.87	0.068	0.45	-0.001	0.99	0.047	0.60
Pollen allergy	-0.017	0.85	-0.199	0.03	0.089	0.33	0.082	0.36	-0.101	0.27	-0.19	0.04	0.092	0.31
Spearman's correlation test was conducted.														

**Table 4:** Speaman's correlation for each variable of the 124 participants BMI: body mass index; FMD: flow-mediated dilatation; PWV: pulse wave velocity; ABI: ankle-branchial index; hs-CRP: high-sensitivity C-reactive protein; ADMA: asymmetric dimethylarginine; NO_2_^-^: nitrite; NO_3_^-^: nitrate; r: the correlation coefficient; p: p-value

Variables	L-arginine		L-citrulline		L-ornithine		L-arginine/ADMA		L-arginine/L-ornithine	
	r	p	r	p	r	p	r	p	ｒ	ｐ
Age	0.08	0.37	0.128	0.16	0.002	0.98	-0.051	0.58	0.032	0.73
Sex	-0.154	0.09	-0.029	0.75	-0.169	0.06	0.005	0.95	0.044	0.62
BMI	0.003	0.97	-0.062	0.50	0.179	0.05	-0.151	0.09	-0.161	0.07
FMD	0.07	0.44	0.131	0.15	0.059	0.51	0.043	0.64	0.010	0.91
PWV	0.013	0.88	-0.039	0.67	0.078	0.39	-0.105	0.25	-0.124	0.17
ABI	0.014	0.88	-0.016	0.86	0.032	0.73	-0.056	0.53	0.017	0.86
Systolic blood pressure	0.085	0.35	0.042	0.64	0.200	0.03	-0.086	0.35	-0.137	0.13
Diastolic blood pressure	0.065	0.47	-0.021	0.81	0.222	0.01	-0.103	0.25	-0.186	0.04
hs-CRP	-0.064	0.48	-0.148	0.10	-0.028	0.76	-0.138	0.13	-0.058	0.52
Arginase 1	-0.02	0.82	0.067	0.46	0.297	0.001	-0.063	0.49	-0.298	0.001
Arginase activity	-0.109	0.23	-0.026	0.77	0.211	0.02	-0.14	0.12	-0.309	<0.001
L-arginine	-	-	0.43	<0.001	0.470	<0.001	-	-	-	-
L-ornithine	0.470	<0.001	0.405	<0.001	-	-	0.226	0.01	-	-
L-citrulline	0.430	<0.001	-	-	0.405	<0.001	0.272	0.002	0.007	0.92
ADMA	0.253	0.005	0.144	0.11	0.250	0.005	-	-	-0.054	0.55
L-arginine/ADMA	-	-	0.272	0.002	0.226	0.01	-	-	-	-
NO_2_^−^	0.177	0.05	0.164	0.07	0.114	0.21	0.075	0.41	0.045	0.62
NO_3_^−^	-0.063	0.49	-0.025	0.78	-0.168	0.06	-0.075	0.41	0.097	0.29
Alcohol consumption	0.065	0.47	-0.107	0.24	0.025	0.78	-0.021	0.82	0.046	0.61
Exercise	0.070	0.44	0.030	0.74	0.005	0.95	0.014	0.88	0.073	0.42
Pollen allergy	-0.157	0.08	-0.191	0.03	-0.126	0.17	-0.051	0.58	0.023	0.80
Spearman's correlation test was conducted.										

FMD was negatively correlated with age, PWV, NO_3_^−^, and alcohol consumption. PWV was positively correlated with age, BMI, blood pressure, hs-CRP, and alcohol consumption, and negatively correlated with FMD. Arginase 1 values showed a very high correlation coefficient with arginase activity, confirming the measurement system reliability. Arginase activity positively correlated with BMI and negatively with NO_2_^−^. ADMA was positively correlated with BMI and systolic pressure. The L-arginine/ADMA ratio correlated positively with ornithine and L-citrulline. NO_2_^−^ was negatively correlated with hs-CRP and arginase activity. Age, BMI, and blood pressure for FMD and PWV corroborate with previous reports [14]. However, there is no consensus on the correlations with alcohol consumption, NO_3_^−^; thus, a multiple regression analysis to explore these correlations is warranted. The correlations of ADMA with BMI and blood pressure, and the bioavailability index with amino acids, are consistent with a previous report [5]. The inverse correlation between arginase activity and NO_2_^−^ also indicates that arginase competes with NO production by NOS (NO_2_^−^) by consuming L-arginine [6].

Multiple regression analysis with FMD and PWV as objective variables

In the multiple regression analysis of FMD and PWV in the overall study population, PWV showed a significant negative association with FMD, while SBP and age showed significant positive associations with PWV. However, factors contributing to the biological availability of NO were not significantly associated with either FMD or PWV (Table 5).

**Table 5:** Factors affecting FMD or PWV in the multiple regression analysis of 124 participants β: standardized partial regression coefficient; p: p-value

Objective variables	Explanatory variables	β	p	Adjusted R^2^
					FMD	PWV	-0.283	0.002	0.072
PWV	Systolic blood pressure	0.568	<0.001	0.586
	Age	0.362	<0.001	
Explanatory variables included in the objective variable are flow-mediated dilation (FMD), pulse wave velocity (PWV), arginase 1 or arginase activity, age, sex, body mass index, ankle-branchial index, high-sensitivity C-reactive protein, systolic blood pressure, asymmetric dimethylarginine, L-arginine, L-ornithine, L-citrulline, nitrite, nitrate, alcohol consumption, exercise habits, and pollen allergy. To avoid multicollinearity, either arginase 1 or arginase activity was used as an explanatory variable. Asymmetric dimethylarginine was converted to the power of 1.7.				

Based on these results, subgroup analyses stratified by age and mean SBP were conducted (Tables 6-7). In the group with SBP exceeding 120 mmHg, NO_3_^-^ showed a negative association with PWV.

**Table 6:** Factors affecting PWV in the multiple regression analysis, stratified by age 58, in 124 participants β: standardized partial regression coefficient; p: p-value

Stratification	Objective variables	Explanatory variables	β	p	Adjusted R^2^
						Age < 58	PWV	Systolic blood pressure	0.709	<0.001	0.494
Age ≥ 58	PWV	Systolic blood pressure	0.585	0.001	0.33
Explanatory variables included in the objective variable are flow-mediated dilation, pulse wave velocity (PWV), arginase 1 or arginase activity, age, sex, body mass index, ankle-branchial index, high-sensitivity C-reactive protein, systolic blood pressure, asymmetric dimethylarginine, L-arginine, L-ornithine, L-citrulline, nitrite, nitrate, alcohol consumption, exercise habits, and pollen allergy. To avoid multicollinearity, either arginase 1 or arginase activity was used as an explanatory variable. Asymmetric dimethylarginine was converted to the power of 1.7.					

**Table 7:** Factors affecting PWV in the multiple regression analysis, stratified by systolic blood pressure of 120 mmHg, in 124 participants β: standardized partial regression coefficient; p: p-value

Stratification	Objective variables	Explanatory variables	β	p	Adjusted R^2^
Systolic blood pressure < 120	PWV	Age	0.471	<0.001	0.354
		FMD	-0.249	0.03	
Systolic blood pressure ≥ 120	PWV	Age	0.509	<0.0001	0.273
		NO_3_^−^	-0.244	0.03	
Explanatory variables included in the objective variable are flow-mediated dilation (FMD), pulse wave velocity (PWV), arginase 1 or arginase activity, age, sex, body mass index, ankle-branchial index, high-sensitivity C-reactive protein, systolic blood pressure, asymmetric dimethylarginine, L-arginine, L-ornithine, L-citrulline, nitrite, nitrate (NO_3_^−^), alcohol consumption, exercise habits, and pollen allergy. To avoid multicollinearity, either arginase 1 or arginase activity was used as an explanatory variable. Asymmetric dimethylarginine was converted to the power of 1.7.					

**Figure 2:**
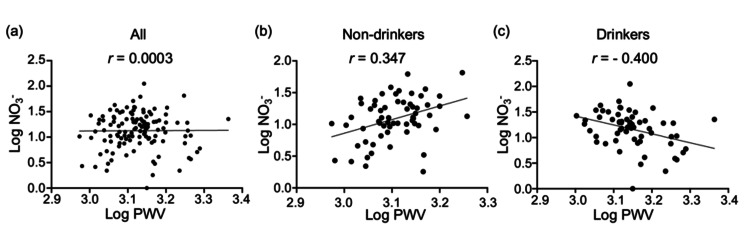
Scatter plots showing the correlation between log-transformed NO3- levels and pulse wave velocity (PWV): (a) all participants, (b) non-drinkers, and (c) drinkers Among the subjects of this study, there is a significant positive correlation between blood NO_3_^-^ levels and PWV in the group with no alcohol consumption, but this relationship is reversed in the group with alcohol consumption. The graph reflects the Spearman's correlation coefficient calculated between log-transformed NO_3_^-^ (Log NO_3_^-^) and log-transformed brachial-ankle pulse wave velocity (Log PWV). Panel (a) shows the results for all participants. There is no significant correlation (r = 0.0003, p = 0.997). Panel (b) shows the results for the non-drinkers (r = 0.347, p = 0.0057). Panel (c) shows the results for the drinkers (r = -0.400, p = 0.0013).

Subsequently, multiple regression analyses stratified by alcohol consumption were performed because this is significantly correlated with both FMD and PWV in Table 2. In the non-drinking group, NO_3_^-^ was positively associated with PWV, whereas L-citrulline was negatively associated. In the drinking group, NO_3_^-^ showed a negative association with PWV (Figure 2 and Table 8).

**Table 8:** Factors affecting FMD or PWV in the multiple regression analysis, stratified by alcohol consumption, in 124 participants β: standardized partial regression coefficient; p: p-value

Stratification	Objective variables	Explanatory variables	β	p	Adjusted R^2^
						Non-drinker	FMD	Age	-0.289	0.02	0.068
PWV	Systolic blood pressure	0.613	<0.001	0.630		
	Age	0.277	0.001			
	NO_3_^−^	0.241	0.005			
	L-citrulline	-0.222	0.007			
Drinker	FMD	PWV	-0.263	0.04	0.054
	PWV	Systolic blood pressure	0.469	<0.001	0.639
		Age	0.411	<0.001	
		NO_3_^−^	-0.274	0.001	
Explanatory variables included in the objective variable are flow-mediated dilation (FMD), pulse wave velocity (PWV), arginase 1 or arginase activity, age, sex, body mass index, ankle-branchial index, high-sensitivity C-reactive protein, systolic blood pressure, asymmetric dimethylarginine, L-arginine, L-ornithine, L-citrulline, nitrite, nitrate (NO_3_^-^), alcohol consumption, exercise habits, and pollen allergy. To avoid multicollinearity, either arginase 1 or arginase activity was used as an explanatory variable. Asymmetric dimethylarginine was converted to the power of 1.7.					

Moreover, because BMI was significantly correlated with PWV, multiple regression analysis stratified by the mean BMI demonstrated that arginase activity was positively and significantly associated with PWV in the group with BMI <23 (Table 9).

**Table 9:** Factors affecting PWV in the multiple regression analysis, stratified by BMI, in 124 participants β: standardized partial regression coefficient; p: p-value

Stratification	Objective variables	Explanatory variables	β	P	Adjusted R^2^
BMI < 23	PWV	Systolic blood pressure	0.617	<0.001	0.723
		FMD	-0.214	0.007	
		Age	0.253	0.004	
		Arginase activity	0.196	0.011	
BMI ≧ 23	PWV	Systolic blood pressure	0.55	<0.001	0.576
		Age	0.406	<0.001	
Explanatory variables included in the objective variable are flow-mediated dilation (FMD), pulse wave velocity (PWV), arginase 1 or arginase activity, age, sex, body mass index, ankle-branchial index, high-sensitivity C-reactive protein, systolic blood pressure, asymmetric dimethylarginine, L-arginine, L-ornithine, L-citrulline, nitrite, nitrate, alcohol consumption, exercise habits, and pollen allergy. To avoid multicollinearity, either arginase 1 or arginase activity was used as an explanatory variable. Asymmetric dimethylarginine was converted to the power of 1.7.					

Multiple regression analysis with L-arginine/ADMA ratio and NO_2_^−^ as objective variables

In the context of atherosclerotic changes in healthy, non-smoking middle-aged adults, BMI is likely to serve as a simple and objective indicator that reflects unhealthy lifestyle habits [15]. Therefore, multiple regression analyses stratified by BMI were conducted using NO-related factors - specifically, the well-known and straightforward indicators NO_2_^-^ and the L-arginine/ADMA ratio - as dependent variables (Table 5). L-citrulline was positively associated with NO_2_^−^ in the group with a BMI below 23, as was hs-CRP for the L-arginine/ADMA ratio. Among those with a BMI of 23 or above, arginase activity was negatively associated with NO_2_^−^, and L-citrulline was positively associated with the L-arginine/ADMA ratio. Notably, in the group with a BMI greater than 23, PWV was adversely correlated with the L-arginine/ADMA ratio (Table 10).

**Table 10:** Factors affecting NO2−, ADMA, L-arginine/ADMA, arginase 1, or arginase activity in the multiple regression analysis, stratified by BMI, in 124 participants β: standardized partial regression coefficient; p: p-value

Stratification	Objective variables	Explanatory variables	β	p	Adjusted R^2^
BMI < 23	NO_2_^−^	L-citrulline	0.349	0.008	0.122
	L-arginine/ADMA	hs-CRP	-0.335	0.011	0.112
BMI ≧ 23	NO_2_^−^	Arginase activity	-0.426	<0.001	0.169
	L-arginine/ADMA	L-citrulline	0.279	0.018	0.128
		PWV	-0.277	0.019	
Explanatory variables included in the objective variable are flow-mediated dilation (FMD), pulse wave velocity (PWV), arginase 1 or arginase activity, age, sex, body mass index (BMI), ankle-branchial index, high-sensitivity C-reactive protein (hs-CRP), systolic blood pressure, asymmetric dimethylarginine (ADMA), L-arginine, L-ornithine, L-citrulline, nitrite (NO_2_^-^), nitrate, alcohol consumption, exercise habits, and pollen allergy. To avoid multicollinearity, either arginase 1 or arginase activity was used as an explanatory variable. Furthermore, when the L-arginine/ADMA ratio was used as the objective variable, L-arginine and ADMA were excluded from the explanatory variables. ADMA was converted to the power of 1.7.					

The significant association of each amino acid with these factors related to NO bioavailability is fully consistent with previous reports [6,8,16].

## Discussion

This study has proven the hypothesis that some of the factors related to the biological availability of NO reflect the early stages of atherosclerotic changes in healthy individuals in middle age. This finding may be applicable as an objective indicator for preventive intervention against atherosclerotic changes and vascular endothelial dysfunction in non-smoking healthy middle-aged individuals with no significant abnormalities in blood pressure, glucose tolerance, or lipids.

Overall, multiple regression analysis demonstrated that age is a strong risk factor for vascular function and that a lower SBP, even within the normal range, is more preferred (Table 5). These results are consistent with those of previous large cohort studies [1] and seem to underscore the validity of the present study. Paradoxically, the failure of NO bioavailability-related factors to reveal significant associations with vascular function in the overall analysis could be attributed to the selection of legitimate healthy participants through a stringent exclusion criterion.

We conducted an exploratory subgroup analysis according to lifestyle factors and physiological tests, successfully determining potential candidates for NO bioavailability-related factors that act as preventive intervention indicators targeting vascular dysfunction. Specifically, higher plasma L-citrulline levels in the group abstaining from alcohol were associated with lower PWV, indicating reduced arterial stiffness (Table 8). Additionally, higher L-citrulline levels were correlated with increased NO_2_^−^ levels in the low-BMI group and an increased L-arginine/ADMA ratio in the high BMI group (Table 9). These results indicate that interventions aimed at increasing L-citrulline may more effectively reduce arterial stiffness compared to L-arginine supplementation, partially supporting previous reports [16].

Notably, several stratified analyses revealed a significant association between plasma NO_3_^−^ levels and PWV. This is likely because NO_3_^−^ is converted to NO through the nitrate-nitrite-NO pathway by oral and gut microbiota, stomach pH, and blood enzymes [8,17]. An important aspect of this pathway is that it improves NO bioavailability, independent of endothelial NOS activity. Notably, participants were prohibited from consuming foods that could affect plasma NO_3_^−^ levels 24 hours before blood sampling. Therefore, the reversal of the association between PWV and alcohol consumption indicates that NO_3_^−^ conversion to NO may undergo dynamic changes in response to alcohol intake, warranting further investigation. In fact, studies report that even moderate alcohol consumption habits of 20 g or less per day alter the oral microbiota compared to non-drinkers, suggesting a potential change in NO3- metabolism [17,18]. Alternatively, from the perspective of NO_3_^−^, having direct adverse effects on vascular function, NO_3_^−^ could be measured as a metabolite of peroxynitrite (ONOO-) [19]. However, direct measurement is challenging because of the high reactivity of ONOO- with proteins. This hypothesis could be partially addressed by measuring its reaction product, nitrotyrosine, which is a crucial focus for future research [20]. Excessive alcohol consumption has impaired endothelial function and exacerbated atherosclerosis, but the effects of moderate alcohol intake (<20 g) on vascular function, as investigated in this study, remain a subject of ongoing debate [21,22]. Moreover, the Japanese population, the participants of this study, is a racial group in which the activity of ALDH2, which detoxifies aldehydes, an important alcohol metabolite, varies greatly depending on genetic polymorphisms, and the relationship with aldehydes related to vascular function must be considered separately [23]. A growing number of clinical studies have recently demonstrated that a high intake of vegetables rich in NO_3_^−^ increases blood NO_2_^−^ (i.e., blood NO) along with blood NO_3_^−^ and thus may improve FMD and PWV [17,24]. Based on the present study results, an intervention study stratified by groups that moderately drink alcohol and groups that do not drink at all may render the effects of such a study more explicit.

Finally, multiple regression analysis was conducted using BMI, an easily used intervention indicator for healthy participants, as the stratification variable and NO bioavailability indicators as the objective variables. In addition to L-citrulline, hs-CRP was significantly associated with L-arginine/ADMA ratio in the low-BMI group, and arginase activity with NO_2_^−^ in the high-BMI group. hs-CRP is a sensitive marker reflecting low levels of chronic inflammation, and its association with vascular dysfunction has been previously observed [25].

The association between arginase activity and NO_2_^−^ in the high-BMI group is almost consistent with previous reports [14], and the decrease in NO_2_^−^ due to elevated arginase directly reflects the mechanism of reduced vascular function, which may be useful as an indicator of the improvement effects of the intervention [26]. The findings indicate that PWV and the L-arginine/ADMA ratio are negatively correlated in the high-BMI group, suggesting that the blood L-arginine/ADMA ratio may be a defining indicator of vascular extensibility in obese, healthy adults. These blood indicators may open an alternative approach to easily reflect arterial function.

The limitations of this study are as follows. Although participants were instructed to refrain from consuming meals containing more than 2000 µg/g of nitrate on the day prior to the study visit, the results may also have been influenced by other factors such as actual dietary records, oral hygiene practices (e.g., frequency and method of mouth rinsing), and the composition of the oral microbiota. These factors were not assessed in the present study and represent important considerations for future research. In the present stratified analysis, participants with a daily alcohol intake of ≤20 g were further subdivided into two groups for comparison. However, the European Food Safety Authority (EFSA) defines low-risk alcohol consumption as up to 20 g/day for men and 10 g/day for women, highlighting known sex differences in susceptibility to alcohol-related health effects. This sex-specific threshold was not considered in the current analysis, which may have influenced the observed associations. Furthermore, although the study applied specific exclusion criteria, important factors such as circadian rhythm patterns, which may influence NO metabolism and vascular function, were not assessed or controlled for in participant selection [27]. As such, the inclusion framework did not fully account for physiological variables that could potentially impact the outcomes. This study did not measure NO itself or important oxidative stress indicators as related factors, which should be addressed in future research. The results of the stratified analysis are secondary endpoints, and that data is insufficient to verify the conclusions. The study is a single Japanese study and cannot be simply compared with Western studies; it is a cross-sectional study, and the causal relationship is not known. To pursue the target factors obtained in this study in more detail, conducting a cohort study with several samples from several ethnic groups is necessary. However, these studies, conducted on nearly all middle-aged healthy participants, may be valuable as pilot studies to identify new targets for the prevention of vascular dysfunction.

## Conclusions

This cross-sectional exploratory study demonstrated that specific NO-related factors in the blood, namely L-citrulline, arginase activity, and nitrate (NO_3_^-^), may serve as early indicators of vascular changes in healthy, middle-aged, non-smoking individuals. Although these markers did not show consistent associations with vascular function in the entire population, stratified analyses revealed meaningful subgroup-specific relationships. Notably, higher plasma L-citrulline levels were associated with reduced arterial stiffness among alcohol consumers and with favorable NO bioavailability profiles depending on BMI. Furthermore, NO_3_^-^ exhibited opposite associations with PWV depending on alcohol intake, suggesting a potential interaction between lifestyle factors and NO metabolism. These findings imply that stratification by lifestyle or physiological characteristics may be critical in identifying individuals who are most responsive to NO-related interventions.

Importantly, this study underscores the potential of NO-related biomarkers as practical, modifiable targets for early preventive strategies against vascular dysfunction. Factors such as L-citrulline and arginase activity are particularly attractive given their amenability to nutritional or supplemental modulation. While the cross-sectional design limits causal inference, the results provide a foundation for future longitudinal or interventional studies aimed at validating these biomarkers and elucidating their role in vascular health. Tailored preventive strategies based on NO bioavailability may offer a more individualized approach to atherosclerosis prevention in otherwise healthy adults, particularly those with subclinical vascular changes.
